# The Use of Bone Density Scan in Monitoring Treatment Response in Patients Diagnosed with Osteoporosis: A Retrospective Cohort Study

**DOI:** 10.1155/2023/2160346

**Published:** 2023-10-23

**Authors:** Mohammed O. Ibrahim, Ahmad Kolleri, Amel Ginawi

**Affiliations:** ^1^College of Medicine, Mohammed Bin Rashid University of Medicine and Health Sciences, Dubai, UAE; ^2^Department of Rheumatology, Mediclinic City Hospital, Dubai, UAE

## Abstract

Osteoporosis is characterized as a metabolic bone disease defined by low bone mineral density (BMD) and bone tissue degeneration, particularly a reduction in the number of trabeculae and a drop in cortical bone thickness, and a rise in porosity, which is mainly due to an imbalance between bone resorption and formation. As a result, it increases bone fragility, and the susceptibility to fracture increases, especially among the elderly. The objective is to assess the effectiveness of dual-energy X-ray absorptiometry (DXA) scan in monitoring the response to osteoporosis treatment and compare the scan's response to different osteoporosis treatments. This retrospective cohort study included 51 adults selected from 300 patients diagnosed with osteoporosis based on World Health Organization (WHO) diagnostic criteria of a *T*-score of -2.5. Data were acquired from the electronic medical records between 2016 and 2019 from a private hospital in Dubai, United Arab Emirates (UAE). The study included sociodemographic characteristics, biomedical parameters, comorbidities, history of fracture, medications, laboratory, and DXA scan results. Ninety-four percent of the patients were females; the mean (±SD) age was 58.1 ± 11.5 years. Most patients were expatriates (84.3%), of which Asian ethnicity was 66.7%. The mean (±SD) duration of osteoporosis was 2.82 ± 1.8 years. Eleven (21.6%) patients had a history of fragility fracture. Ninety-six percent of the patients had vitamin D deficiency. One-third (29.4%) of the patients had hyperparathyroidism. Alendronate/cholecalciferol, received by nine patients (17.6%), showed a significant improvement (*p* = 0.018) in the BMD of the femoral neck among the study group. In conclusion, the DXA scan as a monitoring tool has shown a significant improvement in the BMD of the femoral neck among patients taking alendronate/cholecalciferol treatment compared to other medications.

## 1. Background

Osteoporosis is characterized as a metabolic bone disease defined by low BMD and bone tissue degeneration, particularly a reduction in the number of trabeculae and a drop in cortical bone thickness, and a rise in porosity, which is mainly due to an imbalance between bone resorption and formation [1]. As a result, it increases bone fragility, and the susceptibility to fracture increases, especially among the elderly [1]. Diagnosis of osteoporosis is chiefly based on a *T*-score, reflecting the BMD of the neck of the femur bone and lumbar spine [2]. The WHO has set several scores for osteoporosis; these scores are based on SD and are expressed as *T*-scores [2]. To estimate the *T*-score, the recommended reference range uses measurements of the femoral neck in Caucasian females between the age of 20-29 years, which is based on guidelines from the National Health and Nutrition Examination Survey (NHANES) III reference database [3]. The *T*-score is the difference in SD of the measured BMD from the mean, gender, and race adjusted, BMD of young adults in a population [4]. According to the WHO, patients with a *T* − score < −2.5 SD are considered osteoporotic patients [2]. Osteoporosis is considered a significant noninfectious disease and the most prevalent bone disorder, affecting 1 : 3 women and 1 : 5 men above the age of 50 globally [5, 6]. The prevalence of low BMD is more eminent in the Middle East and North African (MENA) countries in comparison to Western countries [7]. Additionally, vitamin D deficiency is prevalent in the MENA region, which could be a causative factor in osteoporosis [7, 8].

In the UAE, the prevalence of osteoporosis is gradually increasing due to increased life expectancy due to advanced healthcare services [9]. According to a 2019 WHO report, life expectancy in the UAE at birth for women was 78 years and 75 years for men [9]. In 2011, it was shown that 7% of the UAE population were 50 years or older, whereas fewer than 1% were 70 years old [10]. By the year 2050, 12% of the UAE population is predicted to be over 50 years of age, while 2% will be 70 years or over [10]. As a result, osteoporosis prevalence is expected to grow in the coming years in the UAE. Osteoporosis is usually diagnosed using a common densitometric technique known as the DXA, and even though the DXA scan is considered the gold standard in diagnosing osteoporosis, it is worth mentioning that there are other tools used for testing the BMD [11]. The other BMD testing options include quantitative computed tomography (QCT), mainly used as a research tool that measures the hip and/or spine [12]. The second option is the peripheral QCT (pQCT), which measures the lower and upper limbs, including the tibia and forearms, and can produce detailed microstructural imaging, illustrating an image similar to a virtual bone biopsy [13]. Another option used to measure and diagnose osteoporosis is called quantitative ultrasound (QUS); however, the use of QUS is limited to the heels and fingers only [12]. Therefore, its usage in clinics is insignificant.

Pharmacological treatment options are divided into antiresorptive agents, which include bisphosphonates, monoclonal antibodies, and estrogen that suppress bone resorption, and anabolic agents, such as teriparatide and abaloparatide, that promote bone formation [14]. Examples of bisphosphonates would be risedronate, ibandronate, zoledronate, etidronate, tiludronate, pamidronate, and alendronate, which are used in the treatment of osteoporosis in postmenopausal women, and denosumab would be an example of monoclonal antibodies [14]. Patients also need to be aware of the importance of lifestyle changes that include risk factors such as age, gender, smoking behavior, and alcohol intake [15]. Also, attention to sufficient consumption of particular nutrients is critical in osteoporotic patients [15]. The guidelines usually recommend an intake of at least 1000 mg/day of calcium and 800 IU of vitamin D in the management of patients with osteoporosis [16]. In addition, fracture risk assessment is an important diagnostic and screening tool that aids in diagnosing osteoporosis [17]. For example, FRAX, a fracture risk assessment tool, was created to determine the likelihood of a hip fracture and a significant osteoporotic fracture occurring in 10 years [17]. It depends on particular patient models that consider both the femoral neck's BMD and potential risks related to clinical risk factors [17]. Since most of the studies are about the effectiveness of the DXA scan as a diagnostic tool, this study is essential because it will assess the accuracy and efficacy of the DXA scan as a monitoring tool in osteoporosis treatment.

### 1.1. Study Aim

Assessing the role and the effectiveness of DXA scan in monitoring the response and changes in BMD (change in *T*-score) following osteoporosis treatment.

### 1.2. Study Objectives

1. To assess the effectiveness of DXA scan in monitoring the response to osteoporosis treatment
2. Comparing the DXA scan's (*T*-score) response to different treatments of osteoporosis

### 1.3. Research Question

What is the DXA scan's efficacy in monitoring patients on antiosteoporotic drugs and responsiveness to changes in BMD in the last four years from 2016 to 2019?

### 1.4. Research Hypothesis

The DXA scan is an efficient tool in monitoring the response to the treatment of osteoporosis.

## 2. Methods

### 2.1. Study Design, Setting, and Participants

This retrospective cohort study was conducted at Mediclinic City Hospital, a private hospital in Dubai, UAE. This study enrolled adult patients (over the age of 18) who were previously diagnosed with primary and secondary osteoporosis. All patients under the age of 18 were excluded from this study.

Informed consent was waived by the Mohammed Bin Rashid University of Medicine and Health Sciences (MBRU) Institutional Review Board as this retrospective study used electronic medical record data that contained no personal identifiers. All methods were carried out in accordance with relevant guidelines and regulations. Ethical approval was granted by the Mohammed Bin Rashid University of Medicine and Health Sciences (MBRU) Institutional Review Board.

### 2.2. Variables

The exposure being studied is osteoporosis medications, and the outcome investigated is the enhancement of the BMD. Factors considered as potential confounders are age, sex, body mass index (BMI), vitamin D deficiencies, medications, smoking, postmenopausal women, and scans occurring on multiple different machines. Furthermore, in terms of diagnostic criteria, osteoporosis is diagnosed when the *T*-score is ≤ -2.5 at the lumbar spine, femur neck, or hip using the DXA scan [2].

### 2.3. Data Sources/Measurement

Data for analysis was derived from the Mediclinic City Hospital electronic medical records between the 1st of January 2016 and the 31st of December 2019, accessible through the hospital's database. Data measurement was conducted through a structural format that includes sociodemographic characteristics (age, gender, nationality, ethnicity, and smoking behavior), biomedical parameters (weight, height, BMI, and duration of osteoporosis), dietary habits (calcium and caffeine intake), comorbidities (diabetes mellitus, rheumatoid arthritis, ischemic heart disease, hyperparathyroidism, hyperthyroidism, and celiac disease), history of fracture, medications (antiosteoporosis medications, steroids, proton pump inhibitors (PPIs), aromatase inhibitors, antirheumatic medications, and vitamin D supplements), lab results (vitamin D, parathyroid hormone, thyroid stimulating hormone, calcium, and phosphate levels), and DXA scan results (i.e., *T*-score and percentage change in BMD).

### 2.4. Bias

In addition to potential confounders, retrospective cohort studies may suffer from selection bias since a retrospective cohort study starts after all disease cases have occurred. Furthermore, a structured format was prepared to collect similar and equal information from all the participants to avoid data collection bias.

### 2.5. Study Size

The estimated study (number of patients) was 51 adults selected from 500 patients diagnosed with osteoporosis based on WHO diagnostic criteria of a *T*-score of -2.5, extracted from the electronic medical records at Mediclinic City Hospital.

### 2.6. Quantitative Variables

Age, BMI, and number of DXA scans are a few quantitative variables that were considered, and some were considered as potential confounders.

### 2.7. Statistical Methods

Data were entered in a Microsoft Excel spreadsheet and were analyzed using Statistical Product and Services Solutions (SPSS) 24.0 [18]. Since the *T*-score measurement of the DXA scan is presented as mean and SD, an independent-sample *t*-test was used to compare means between the before and after interventions and detect any significant difference. The outcome was the change in *T*-scores, which is a continuous variable. Moreover, a one-sample *t*-test was used to detect the mean, SD, and *p* value of the femoral neck *T*-score change between pre- and posttreatment; the same was conducted for the lumbar spine *T*-score values. In addition, a paired-sample *t*-test was used to correlate between the *T*-scores before and after treatment in correlation with other medications and comorbidities. Moreover, as the change depends on the patient's age and gender, a multivariable regression analysis was used to determine any correlation between the means before and after the intervention. The change in *T*-scores is the independent variable.

### 2.8. Power and Sample Size Calculation

The power calculation is not needed for retrospective time-frame studies. The sample size available is estimated to be 51 participants, extracted from the electronic medical records at Mediclinic City Hospital.

### 2.9. Expected Outcomes

If the changes in *T*-scores are statistically significant, there should be a significant improvement in the BMD of patients who received osteoporosis treatment for at least one year. In that case, DXA scans should be able to accurately pick up any positive or negative BMD changes, thus making it an efficient and accurate tool to monitor the response to osteoporosis treatment.

## 3. Results

This study includes a total of 51 patients with osteoporosis who were recruited from January 2016 to December 2019. Ninety-four of the patients were females. The patients' mean age (±SD) was 58.1 ± 11.5 years, with a minimum of 32 and a maximum of 88. Most of the patients were expatriates (84.3%), and from which the Asian ethnicity was 66.7%. The mean (±SD) duration of osteoporosis was 2.82 ± 1.85 years. Nonsmokers comprised 76.5% of the patients; 54.9% consumed more than 2 cups of coffee daily, and 72.5% took calcium supplements. Out of the 51 patients, 11 (21.6%) patients have a history of fracture. It was evident that 96.1% of the patients had vitamin D deficiency. The age categories of the study sample follow a normal distribution, with the highest frequency of patients being between 51 and 60 years old and the lowest frequency between 31 and 40 years old. Around half of the study group were categorized as having normal weight (47.1%). On the other hand, about one-third were overweight (27.5%), one-third were obese (23.5%), and one patient was underweight (2%) (Table 1).

Table 2 shows that almost one-third of the study group suffered from diabetes, hyperparathyroidism, and rheumatoid arthritis. 35.3% of patients have diabetes, 29.4% have hyperparathyroidism and rheumatoid arthritis, 5.9% have ischemic heart disease, and 3.9% have celiac disease. 96.1% were diagnosed with vitamin D deficiency.

Table 3 shows that almost half of the study population was prescribed denosumab (49%), followed by alendronate/cholecalciferol (17.6%), and alendronate (13.7%). In contrast, raloxifene and zoledronate were prescribed in only 1% of the study group. In addition, (21.6%) of the study group were not taking antiosteoporotic drugs, as they were prescribed vitamin D and calcium supplementation instead, as shown in Table 4 under other medications. Most of the study group (72.5%) were prescribed vitamin D supplements. In addition, twenty-nine patients (56.9%) were taking PPIs, followed by steroids (41.2%).

Table 4 shows the effectiveness of different medications on the mean change of the *T*-score of the femoral neck. As shown, alendronate/cholecalciferol was received by nine patients (17.6%) of the study group with a mean *T*-score value (SD) of -0.377 (.506) with a *p* value of 0.018. On the other hand, denosumab, which was given to 25 patients (49%), with a mean (SD) *T*-score of -0.136 (0.476), had a *p* value of 0.528.

**Figure 1:**
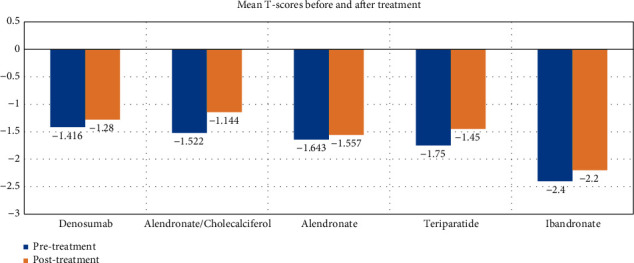
Mean *T*-scores of the femoral neck pretreatment and posttreatment according to medication between 2016 and 2019.

Figure 1 shows the mean *T*-scores in the femoral neck before and after treatment according to medication. Alendronate/cholecalciferol shows the most significant increase in mean *T*-score from -1.522 before treatment to -1.144 after treatment.

Table 5 shows the effectiveness of different medications on the mean change of the *T*-score of the lumbar spine. As shown, denosumab was given to 25 patients (49%) of the study group with a mean *T*-score value (SD) of -0.052 (.686) with a *p* value of 0.367. There are multiple reasons why none of the medications showed a statistically significant *p* value, which is discussed in the discussion section of this study.

Table 6 shows the correlation of different comorbidities such as rheumatoid arthritis, diabetes mellitus, hyperparathyroidism, vitamin D deficiency, ischemic heart disease, celiac disease, and smoking in relation to the change of the *T*-score before and after treatment of the femoral neck and lumbar spine. As shown in the table, hyperparathyroidism in the femoral neck had a *p* value of 0.003 and 0.008 in the lumbar spine. In addition, patients with vitamin D deficiency had a *p* value of 0.057 in the femoral neck, and patients with celiac disease had a *p* value of 0.049 in the femoral neck.

Table 7 shows the correlation of other medications taken by the study group, such as steroids, PPIs, aromatase inhibitors, antirheumatic medications, and vitamin D supplementation, with relation to the change of the *T*-score before and after treatment in the femoral neck and lumbar spine. As shown in the table, patients supplementing with vitamin D had a *p* value of 0.008 in the femoral neck and 0.185 in the lumbar spine.

## 4. Discussion

This study reported a significant increase in BMD over four years on alendronate/cholecalciferol at the femoral neck in adult patients previously diagnosed with osteoporosis. In this study, patients preferred denosumab (monoclonal antibody) in comparison to alendronate/cholecalciferol (bisphosphonate) because of the inconvenience of the latter. The latter should be taken in the morning and evening at least thirty minutes before meals, with enough water; the patient should stay upright for at least half an hour following intake to avoid esophagitis. On the other hand, denosumab is given subcutaneously as a single dose once every six months, which the treating physician administers in the clinic. This serves to explain why patients prefer to avoid the inconvenience of alendronate/cholecalciferol, which has led to a significantly decreased number of patients on alendronate/cholecalciferol (17.6%) in comparison to denosumab (49%).

The study sample had a high prevalence of diagnosed vitamin D deficiency. This may be due to the high prevalence of vitamin D deficiency in the UAE [19]. Studies have shown that increased serum 25-hydroxyvitamin D is related to improved BMD until a threshold level [20–22]. 56.9% of the patients took PPIs, 41.2% took steroids, including inhaled corticosteroids, and 9.8% were on aromatase inhibitors. Glucocorticoids can cause bone resorption and predispose to osteoporosis [23].

Moreover, inhaled corticosteroid use has been linked with increased osteoporosis risk in COPD patients [24]. PPIs have an FDA-boxed warning for an increased risk of osteoporotic fractures [25]. Aromatase inhibitors are used as a breast cancer therapy in postmenopausal women [26] and can lead to low BMD [27].

The mean age of the study sample was 58.10 years, and females comprised most of the sample at 94.1%. A study (assessing BMI and BMD in patients referred for DXA scans) conducted in the UAE had 87.1% females, and the mean age was 50.5 years [28]. In an Austrian study with osteoporotic women, the mean age was 65.43 years [29]. Estrogen deficiency after menopause is known to cause bone resorption [30].

The prevalence of diagnosed vitamin D deficiency among the study sample was 96.1% compared to a study conducted in the UAE, which had a 74% vitamin D deficiency [31]. Another international study conducted among osteoporotic women showed a high prevalence of vitamin D inadequacy in the Middle East (81.8%) and Asia (71.4%). In contrast, Europe, Latin America, and Australia had a prevalence of 57.7%, 53.4%, and 60.3%, respectively [32]. The mean serum parathyroid hormone (65.95 ± 30.02 pg/mL) was higher in our study than their mean of 30.7 ± 0.3 pg/mL [32].

72.5% of the sample took calcium supplements, and 72.5% took vitamin D supplements compared to 63.3% and 54.5%, respectively, in a Danish study [33]. Calcium and vitamin D supplementation can prevent bone loss and fractures, and therefore, they are recommended even with antiosteoporotic treatment [34]. The Danish paper also reported a prevalence of 20.8% for fragility fractures [33], compared to 21.6% of our sample that reported as having had fragility fractures which is similar to the prevalence of fractures (21.8%) in a Korean cross-sectional study of postmenopausal women [35].

Most of the sample had normal weight (47.1%) while some were overweight (27.5%) or obese (23.5%). Increased BMI is a protective factor against fractures in some sites such as the hip and spine, while others such as the ankles have an increased risk of fracture [36]. About half of the study sample drank more than 2 cups of coffee or tea per day, and 23.5% were smokers compared to a study that assessed risk factors of osteoporosis in the UAE, where 60% of their sample drank more than 2 cups of coffee and 54% were smokers [37]. 35.3% of the sample were diabetic compared to the aforementioned study where 46% of the osteoporotic patients were diabetic [37]. 41.2% and 9.8% of the sample were taking steroids and aromatase inhibitors, respectively, while in that study, 38.0% had taken steroids, and 6.0% had done chemotherapy [37].

Smoking was found to be related to bone loss in the Rotterdam study [38]. A meta-analysis looking at the relationship between diabetes, BMD, and hip fracture risk found that there was an increased risk of hip fractures in both type 1 and type 2 diabetes although there was increased BMD in type 2 diabetes and decreased BMD in type 1 diabetes [39].

Hyperparathyroidism had a prevalence of 29.4%, which is a risk factor for osteoporosis in younger postmenopausal patients, while in elderly women, it may have a protective effect [40]. Rheumatoid arthritis had a similar prevalence where osteoporosis is considered as an extra-articular manifestation of it [41]. Celiac disease had a prevalence of 3.9% among our sample compared to a Czech study which screened osteoporotic and osteopenic patients for celiac disease and found a prevalence of 2% [42].

Due to direct evidence showing improved BMD in selected participants after concluding a five-year treatment plan, it was recommended that bisphosphonates, particularly alendronate and alendronate/cholecalciferol, be used as the first-line treatment option for osteoporosis [43]. Although bisphosphonates are the first-line therapy for osteoporosis, in 2018, it was found that denosumab was prescribed more frequently by rheumatology practices in the United States [44]. This finding is similar to our study, where approximately half of our sample group were given denosumab compared to other medications. Therefore, monitoring patient response to therapy is critical for identifying patients needing therapeutic changes [45]. While there are several techniques to monitor osteoporosis treatment, there needs to be a general agreement on the best method [46]. According to Watts et al., DXA scans are frequently used to evaluate BMD response; however, DXA does not capture the majority of bisphosphonate effectiveness in fracture reduction [47]. In our study, we have found out that alendronate/cholecalciferol is the only bisphosphonate that was captured by DXA and showed significant improvement in BMD. Following up on BMD using a DXA scan is recommended in various published guidelines for monitoring the response to osteoporosis medication.

Nevertheless, no agreement exists on the best monitoring interval or site [48]. In our study, the DXA scan captured improvement in BMD in patients taking alendronate/cholecalciferol; this improvement was seen in the femoral neck compared to the lumbar spine and did not show any changes in a mean treatment duration of 2-3 years. According to the International Society of Clinical Densitometry (ISCD), they recommended repeating BMD testing in the femoral neck and spine when predicted BMD change meets or exceeds the least significant change, which is generally one to two years after therapy begins, with extended intervals after therapeutic effectiveness is established; also, monitoring more regularly is recommended in situations that cause fast bone loss, such as glucocorticoid treatment [49]. On the other hand, the National Osteoporosis Foundation (NOF) recommends that BMD testing be repeated one to two years after starting treatment and every two years, with more repeated testing in certain clinical conditions [17]. The American Association of Clinical Endocrinologists (AACE) recommends that DXA of the lumbar spine and hip be repeated every two years [50].

Furthermore, finally, the American College of Physicians (ACP) advises against monitoring throughout treatment because many women who receive antiresorptive medication experience a reduction in fractures even if their BMD does not improve [51]. This study correlated that the statistical significance and improvement of BMD is evidence of the DXA scan's ability to capture treatment response in particular sites of the bones and with certain medications. The statistical significance of BMD changes for fracture risk reduction and prevention being monitored by DXA scan is still controversial, and there is minimal evidence that BMD is a valid indicator of fracture risk reduction. A study by Bruyere et al. conducted on patients treated with strontium ranelate showed a correlation between changes in the femoral neck BMD and reduced fracture risk [52]. This data is similar to our finding as alendronate/cholecalciferol was the only medication that has shown statistically significant improvement in BMD. All these findings imply that the need to measure BMD changes to monitor osteoporosis treatment using DXA may be drug-dependent. The DXA scan effectively monitors BMD in patients taking alendronate/cholecalciferol; however, other monitoring modalities should be used for other medications. An informed patient about the longevity of alendronate/cholecalciferol and how it improves the BMD, in the long run, could be a reason to accept the inconvenience of taking it before meals and maintaining an upright position for half an hour weekly.

This study had several limitations. A sample size of 51 patients was selected from a single private hospital in the emirate of Dubai, thus limiting generalizability to other hospitals, emirates, or neighboring countries. DXA scans of patients in the study sample were conducted on different machines. Future research may want to compare the DXA scan with other osteoporosis monitoring tools such as bone turnover markers, and parametric Electric Impedance Tomography (pEIT) scans as a supplementary tool for monitoring osteoporosis treatment.

In conclusion, this study has shown a significant improvement (*p* = 0.018) in the BMD in the femoral neck with patients on alendronate/cholecalciferol treatment. Finally, this study found that BMD was improved only with alendronate in combination with cholecalciferol, where there was no improvement with the administration of alendronate alone. These findings may help inform clinicians about the effectiveness of alendronate/cholecalciferol treatment on BMD if the DXA scan is used as a monitoring tool.

## 5. Limitations

While examining the same patients' scanned results using DXA devices from various companies, caution must be taken as there are trivial differences in major brand parameters [12]. These parameters include voltages and other parameters [12]. Variability in device measurements can be caused by the differences in the coefficient of variation that varies between machines, even from the same manufacturer, variability between technologists, patient positioning on the DXA machine, and other factors [4]. *T*-scores may not be race-adjusted as some companies only adjust to the Caucasian race, while other manufacturers adjust to the subject's race [4]. The small number of our participants is another limitation, as large samples empower the study. In addition, not all patients received the entire treatment duration (2016-2019) during the last four years, as some patients might have been enrolled in, for instance, 2018, while others in 2017. Furthermore, no control group used another modality to monitor BMD improvement other than the DXA scan. Finally, the sample size was taken from the emirate of Dubai, not the whole UAE, and therefore may not be generalizable to other emirates or countries with different demographics.

## Figures and Tables

**Table 1:** Sociodemographic characteristics of osteoporotic patients from 2016 to 2019.

Variables	Number (%)/mean (SD)
Age	58.10 (11.463) years
31 to 40 years	1 (2.0%)
41 to 50 years	13 (25.5%)
51 to 60 years	16 (31.4%)
61 to 70 years	15 (29.4%)
71 to 80 years	4 (7.8%)
81 to 90 years	2 (3.9%)
Duration of osteoporosis	2.82 (1.85) years
Nationality	
Local	8 (15.7%)
Expats	43 (84.3%)
Ethnicity	
Asian	34 (66.7%)
White	15 (29.4%)
Black	1 (2%)
Hispanic	1 (2%)
Gender	
Male	3 (5.9%)
Female	48 (94.1%)
Smokers	
Yes	12 (23.5%)
No	39 (76.5%)
Calcium intake (supplements)	
Yes	37 (72.5%)
No	14 (27.5%)
Coffee intake (>2 cups)	
Yes	28 (54.9%)
No	22 (43.1%)
History of fracture	
Yes	11 (21.6%)
No	40 (78.4%)
BMI	
Underweight	1 (2.0%)
Normal weight	24 (47.1%)
Overweight	14 (27.5%)
Obese	12 (23.5%)

**Table 2:** Frequency of comorbidities among patients diagnosed with osteoporosis between 2016 and 2019.

Comorbidities	Number (%) (*n* = 51)
Diabetes	
Yes	18 (35.3%)
No	33 (64.7%)
Hyperparathyroidism	
Yes	15 (29.4%)
No	36 (70.6%)
Rheumatoid arthritis	
Yes	15 (29.4%)
No	36 (70.6%)
Ischemic heart disease	
Yes	3 (5.9%)
No	48 (94.1%)
Celiac disease	
Yes	2 (3.9%)
No	49 (96.1)
Vitamin D deficiency	
Yes	49 (96.1%)
No	2 (3.9%)

**Table 3:** Antiosteoporosis and other medications used among the study group between 2016 and 2019.

Medications	Total	Number (%)
Denosumab	51	No, 26 (51%) Yes, 25 (49%)
Alendronate/cholecalciferol	51	No, 42 (82.4%) Yes, 9 (17.6%)
Alendronate	51	No, 44 (86.3%) Yes, 7 (13.7%)
Teriparatide	51	No, 49 (96.1%) Yes, 2 (3.9%)
Ibandronate	51	No, 49 (96.1%) Yes, 2 (3.9%)
Denosumab	51	No, 50 (98%) Yes, 1 (2%)
Raloxifene	51	No, 50 (98%) Yes, 1 (2%)
Zoledronate	51	No, 50 (98%) Yes, 1 (2%)
No antiosteoporosis drugs	51	No, 40 (78.4%) Yes, 11 (21.6%)
Other medications		
Vitamin D supplements	51	No, 14 (27.5%) Yes, 37 (72.5%)
PPIs	51	No, 22 (43.1%) Yes, 29 (56.9%)
Steroids	51	No, 30 (58.8%) Yes, 21 (41.2%)
Antirheumatic drugs	51	No, 42 (82.4%) Yes, 9 (17.6%)
Aromatase inhibitors	51	No, 46 (90.2%) Yes, 5 (9.8%)

**Table 4:** Effectiveness of medications on mean change of *T*-score on femoral neck among study group 2016-2019.

Medications	*n* = 51	Mean (SD)	*p* value
Denosumab	No, 26 (51%) Yes, 25 (49%)	-0.0654 (.058) -0.136 (.476)	0.528
Alendronate/cholecalciferol	No, 42 (82.4%) Yes, 9 (17.6%)	-0.040 (.345) -0.377 (.506)	0.018
Alendronate	No, 44 (86.3%) Yes, 7 (13.7%)	-0.102 (.404) -0.085 (.353)	0.919
Teriparatide	No, 49 (96.08%) Yes, 2 (3.92%)	-0.091 (.399) -0.300 (.141)	0.470
Ibandronate	No, 49 (96.08%) Yes, 2 (3.92%)	-0.095 (.399) -0.200 (.282)	0.718

**Table 5:** Effectiveness of different medications on mean change of *T*-score on lumbar spine used between 2016 and 2019.

Medications	*n* = 51	Mean (SD)	*p* value
Denosumab	No: 24 Yes: 25	-0.052 (.686)	0.367
Alendronate/cholecalciferol	No: 40 Yes: 9	-0.0889 (.372)	0.817
Alendronate	No: 42 Yes: 7	-0.328 (.652)	0.398
Ibandronate	No: 47 Yes: 2	-0.200 (.282)	0.886

**Table 6:** Comorbidities with relation to *T*-score before and after treatment of femoral neck and lumbar spine 2016-2019.

Variable	Mean *T* score pretreatment (SD) (femoral neck)	Mean *T*-score posttreatment (SD) (femoral neck)	*p* value	Mean *T*-score pretreatment (SD) (lumbar spine)	Mean *T*-score posttreatment (SD) (lumbar spine)	*p* value
Rheumatoid arthritis	-1.047 (0.917)	-1.067 (1.106)	0.887	-1.113 (1.043)	-1.060 (1.1281)	0.719
Diabetes mellitus	-1.767 (0.856)	-1.661 (0.810)	0.227	-1.678 (1.198)	-1.606 (1.3335)	0.735
Hyperparathyroidism	-2.093 (0.601)	-1.847 (0.699)	0.003	-2.079 (0.955)	-1.829 (0.9466)	0.008
Hypovitaminosis D	-1.645 (0.853)	-1.535 (0.899)	0.057	-1.734 (1.111)	-1.609 (1.1602)	0.197
Ischemic heart disease	-1.933 (1.171)	-2.067 (1.184)	0.456	-0.500 (2.262)	-0.800 (1.9799)	0.374
Celiac disease	-2.050 (0.212)	-1.400 (0.282)	0.049	-2.000 (0.000)	-1.850 (0.0707)	0.205
Smoking	-1.900 (0.436)	-1.833 (0.563)	0.563	-2.045 (0.639)	-1.945 (0.7435)	0.379

**Table 7:** Other medications with relation to change of *T*-score before and after treatment of femoral neck and lumbar spine 2016-2019.

Variable	Mean *T*-score before (femoral)	Mean *T*-score post (femoral)	*p* value	Mean *T*-score before (lumbar)	Mean *T*-score post (lumbar)	*p* value
Steroids	-1.276 (.848)	-1.248 (.702)	.727	-1.176 (1.028)	-0.986 (.992)	.122
PPIs	-1.348 (.936)	-1.245 (.976)	.260	-1.407 (1.156)	-1.200 (1.159)	.069
Aromatase inhibitors	-1.680 (.798)	-1.560 (.743)	.493	-1.540 (1.422)	-1.280 (1.507)	.41
Antirheumatic meds	-0.978 (940)	-0.833 (1.315)	.505	-1.022 (1.039)	-0.800 (1.074)	.329
Vitamin D supplements	-1.689 (.799)	-1.511 (.947)	.008	-1.617 (1.094)	-1.449 (1.193)	.185

## Data Availability

The corresponding author can provide the datasets utilized and/or analyzed within the current study upon reasonable request.

## Abbreviations

DXA: Dual-energy X-ray absorptiometry
BMD: Bone mineral density
WHO: World Health Organization
SD: Standard deviation
MENA: Middle East and North Africa
UAE: United Arab Emirates
QCT: Quantitative computed tomography
QUS: Quantitative ultrasound
BMI: Body mass index
PPIs: Proton pump inhibitors.

## Supplementary Materials

Supplementary Materials Supplementary table S1 shows the mean lab test results among the study group. Vitamin D values had a mean of 36.19 ng/mL (±17.73) with the lowest value being 4.7 ng/mL and the highest 111.0 ng/mL among 45 patients. Calcium levels had a mean of 2.35 mmol/L (±0.15), parathyroid hormone had 65.95 pg/mL (±30.02), thyroid stimulating hormone had 1.94 uIU/mL (±2.13), phosphate had 1.53 mmol/L (±0.80), and alkaline phosphatase had 82.50 U/L (±25.08).
