# Supplementary material for: The Use of Bone Density Scan in Monitoring Treatment Response in Patients Diagnosed with Osteoporosis: A Retrospective Cohort Study
Source: Int J Rheumatol. 2023 Oct 23;2023:2160346. doi: 10.1155/2023/2160346 (PMC10615580; doi:10.1155/2023/2160346)
Supplement: Supplementary Materials — Supplementary table S1 shows the mean lab test results among the study group. Vitamin D values had a mean of 36.19 ng/mL (±17.73) with the lowest value being 4.7 ng/mL and the highest 111.0 ng/mL among 45 patients. Calcium levels had a mean of 2.35 mmol/L (±0.15), parathyroid hormone had 65.95 pg/mL (±30.02), thyroid stimulating hormone had 1.94 uIU/mL (±2.13), phosphate had 1.53 mmol/L (±0.80), and alkaline phosphatase had 82.50 U/L (±25.08). [file 2160346.f1.docx]

Supplementary table S1 shows the mean lab test results among the study group. Vitamin D values had a mean of 36.19 ng/mL (±17.73) with the lowest value being 4.7 ng/mL and the highest 111.0 ng/mL among 45 patients. Calcium levels had a mean of 2.35 mmol/L (±0.15), parathyroid hormone had 65.95pg/mL (±30.02), thyroid stimulating hormone had 1.94 uIU/mL (±2.13), phosphate 1.53 mmol/L (±0.80) and alkaline phosphatase 82.50 U/L (±25.08).

| **Supplementary Table S1: Lab test results among patients diagnosed with osteoporosis between 2016-2019** | | | | | | |
| --- | --- | --- | --- | --- | --- | --- |
| **Lab tests** | **N** | **Reference Range** | **Min.** | **Max.** | **Mean** | **SD** |
| Vitamin D | 45 | <10.0 Deficient  10 - 29.0 Insufficient  30 - 100.0 Sufficient  >100.0 Potential Intoxication | 4.7 | 111.0 | 36.19 | 17.73 |
| Ionized Calcium | 27 | (1.13 - 1.32 mmol/L) | 1.06 | 1.32 | 1.23 | 0.05 |
| Calcium | 41 | (2.10 - 2.55 mmol/L) | 1.92 | 2.84 | 2.35 | 0.15 |
| Parathyroid Hormone | 18 | (15.00 - 65.00 pg/mL) | 25.8 | 140.7 | 65.95 | 30.02 |
| Thyroid Stimulating Hormone | 34 | (0.35 - 4.94 uIU/mL) | .01 | 12.98 | 1.94 | 2.13 |
| Phosphate | 12 | (0.74 - 1.52 mmol/L) | .87 | 3.95 | 1.53 | 0.80 |
| Alkaline Phosphatase | 24 | (40 - 150 U/L) | 47 | 146 | 82.50 | 25.08 |
